# Supplementary material for: CHAMPP4KIDS: Mixed methods study protocol to evaluate acceptability and feasibility of Parenting for Lifelong Health materials in a Canadian context
Source: PLoS One. 2024 Mar 7;19(3):e0298156. doi: 10.1371/journal.pone.0298156 (PMC10919627; doi:10.1371/journal.pone.0298156)
Supplement: Attachment — Submitted filename: Response to Reviewers.docx [file pone.0298156.s001.docx]

PLOS ONE REVISED MANUSCRIPT REQUESTS AND RESPONSES

RESPONSE TO REVIEWERS

Please include the following items when submitting your revised manuscript:

- A rebuttal letter that responds to each point raised by the academic editor and reviewer(s). You should upload this letter as a separate file labeled '**Response to Reviewers'.**
- A marked-up copy of your manuscript that highlights changes made to the original version. You should upload this as a separate file labeled '**Revised Manuscript with Track Change**s'.
- An unmarked version of your revised paper without tracked changes. You should upload this as a separate file labeled '**Manuscript**'.

**Journal requirements:**

When submitting your revision, we need you to address these additional requirements.

1. Please ensure that your manuscript meets PLOS ONE's style requirements, including those for file naming. The PLOS ONE style templates can be found at

<https://journals.plos.org/plosone/s/file?id=wjVg/PLOSOne_formatting_sample_main_body.pdf> and

<https://journals.plos.org/plosone/s/file?id=ba62/PLOSOne_formatting_sample_title_authors_affiliations.pdf>

*We thank the editorial team for their feedback and guidance on formatting issues. We have reformatted the manuscript to conform to PLOS ONE’s style requirements.*

2. We note that you have stated that you will provide repository information for your data at acceptance. Should your manuscript be accepted for publication, we will hold it until you provide the relevant accession numbers or DOIs necessary to access your data. If you wish to make changes to your Data Availability statement, please describe these changes in your cover letter and we will update your Data Availability statement to reflect the information you provide.

*We have updated all of the DOIs in the reference section of our manuscript.*

3. Your ethics statement **should only appear in the Methods** section of your manuscript. If your ethics statement is written in any section besides the Methods, please delete it from any other section.

*We have deleted the ethics statements from all other sections of the manuscript except in the Methods.*

Reviewers' comments:

Reviewer's Responses to Questions

**Comments to the Author**

1. Does the manuscript provide a valid rationale for the proposed study, with clearly identified and justified research questions?

The research question outlined is expected to address a valid academic problem or topic and contribute to the base of knowledge in the field.

Reviewer #1: Partly

Reviewer #2: Yes

2. Is the protocol technically sound and planned in a manner that will lead to a meaningful outcome and allow testing the stated hypotheses?

The manuscript should describe the methods in sufficient detail to prevent undisclosed flexibility in the experimental procedure or analysis pipeline, including sufficient outcome-neutral conditions (e.g. necessary controls, absence of floor or ceiling effects) to test the proposed hypotheses and a statistical power analysis where applicable. As there may be aspects of the methodology and analysis which can only be refined once the work is undertaken, authors should outline potential assumptions and explicitly describe what aspects of the proposed analyses, if any, are exploratory.

Reviewer #1: Yes

Reviewer #2: Yes

3. Is the methodology feasible and described in sufficient detail to allow the work to be replicable?

Descriptions of methods and materials in the protocol should be reported in sufficient detail for another researcher to reproduce all experiments and analyses. The protocol should describe the appropriate controls, sample size calculations, and replication needed to ensure that the data are robust and reproducible.

Reviewer #1: Yes

Reviewer #2: Yes

4. Have the authors described where all data underlying the findings will be made available when the study is complete?

The [PLOS Data policy](https://journals.plos.org/plosone/s/materials-and-software-sharing) requires authors to make all data underlying the findings described in their manuscript fully available without restriction, with rare exception, at the time of publication. The data should be provided as part of the manuscript or its supporting information or deposited to a public repository. For example, in addition to summary statistics, the data points behind means, medians and variance measures should be available. If there are restrictions on publicly sharing data—e.g., participant privacy or use of data from a third party—those must be specified.

Reviewer #1: No

Reviewer #2: No

Because this is a protocol paper, without any data, we selected ‘N/A – No results are reported’ option under data sharing. Please let us know if this is incorrect and we will rectify the situation. Once the project is completed, we will be happy to share data upon request.

5. Is the manuscript presented in an intelligible fashion and written in standard English?

PLOS ONE does not copyedit accepted manuscripts, so the language in submitted articles must be clear, correct, and unambiguous. Any typographical or grammatical errors should be corrected at revision, so please note any specific errors here.

Reviewer #1: Yes

Reviewer #2: Yes

6**. Review Comments to the Author**

Please use the space provided to explain your answers to the questions above and, if applicable, provide comments about issues authors must address before this protocol can be accepted for publication. You may also include additional comments for the author, including concerns about research or publication ethics.

You may also provide optional suggestions and comments to authors that they might find helpful in planning their study.

**Reviewer #1:** Thank you for the opportunity to review this protocol of an important and rigorous study. Overall, I thought the manuscript was clear and organized. There are a couple of areas that I believe would strengthen the manuscript:

*We thank the reviewer for their thoughtful and thorough review which has strengthened the clarity of the paper. We hope our edits address their concerns adequately.*

*1.* p.8 paragraph 2 the authors introduce the constructs that will be assessed to understand acceptability. It would be useful for a bit more information related to how these constructs are defined as this would be helpful in connecting the constructs to the proposed measures.

*Please see lines 248-250which now read: The DOI (64) and the CFIRC(65)contend that the constructs such as complexity, compatibility, trialability, and design of an innovation may facilitate its acceptability and feasibility of an innovation (64, 65). Also: we added DOI to the list of abbreviations.*

*We have also added definitions to Table 1. Please see line 376 Table 1 for construct definitions*

*Adoption of Materials: The likelihood that decision makers will decide to put the innovation in place (65).*

*Compatibility: The degree to which the innovation is perceived to be consistent with socio-cultural values, previous ideas, and/or perceived needs (64).*

*Complexity The degree to which an innovation is difficult to use or understand, its simplicity (64).*

*Trialability: The ability to test the intervention on a small scale in the organization and to be able to reverse course if needed (65).*

*Observability: The degree to which the results of an innovation are visible to potential adopters (64).*

*Visual Appeal: the degree to which the innovation is well designed and presented* (65).

*Understandability: the degree in which the innovation is seen as difficult to understand or use (64).*

*Readability: the degree in which the innovation is seen as difficult to read or comprehend (64).*

2. p. 11 - the authors describe the eligibility criteria - it would be helpful if there was a description of how eligibility will be determined and verified. Particularly because there doesn't seem to be direct contact with participants and study staff until the focus groups. Also, I'm assuming that participants need to be living in Canada, however, I'm not sure if that is an accurate assumption. Therefore, clarification here would be useful.

*Thank you for this point of clarification. We attempt to verify participant eligibility in two ways. First, for the focus groups, we have established partnerships with various community organizations, and they are sharing our recruitment flyers within relevant groups, settings, and directly with clients and members that attend their various programs. Thus, participants who are approached about the study generally meet the eligibility criteria. Eligibility criteria is further confirmed by the Research Assistant, during the consent call using the four pre-screener questions used in the quantitative survey, explained further. Second, for the quantitative survey, we have a pre-screener in place which involves responding to four questions, including**: Do you have a child between the ages of 2-6 years old? Y Do you identify as a member of a racialized group? Are you a newcomer or immigrant to Canada?*

*Please see the following edited sections:*

*Line 323 (tracked changes copy): Are the custodial caregiver, living in Canada, with a child aged 2-6 years at the time of study recruitment. And lines 329-332: listing the 4 screener questions: During the screener, the following questions will be presented to participants: Do you have a child between the ages of 2-6 years old? Have you participated in a parenting program such as Triple P, Nobody's Perfect or Circle of Security within the last 6 years? Do you identify as a member of a racialized group? Are you a newcomer or immigrant to Canada?*

3. Rationale for why the study is collecting CES-D and GAD-7 is needed and how this information informs the acceptability and feasibility evaluation.

*Please see Line 438-444 (tracked changes copy):* *Finally, caregivers will be asked to complete two self-report scales. The first will measure depression called, The Centre for Epidemiologic Studies Depression Scale (CES-D) and the second anxiety The Generalized Anxiety Disorder scale (GAD-7). These two scales are commonly used measures in various parenting program evaluations. As such we will conduct a general comparison to examine if caregiver mental health symptomatology is similar to what is reported in the literature, and whether scores correlated with their perceptions of acceptability and feasibility of the PLH materials.*

Reviewer #2: The study is well designed for the objectives pursued, however I have two concerns that the authors need to consider.

We thank the reviewer for their positive feedback and their concerns outlined below. We provided additional information regarding the recruitment, eligibility criteria and why we have not included a measure of child behavioural problems.

- parents to be eligible have to be proficient in English (which may exclude parents from more vulnerable groups and by feeling excluded and without another answer this may contribute to increase their exclusion);

*We have partnered with various agencies in high priority neighbourhoods to promote advertisement for the study. In addition, several of our partners include Immigration Centres or centres for refugee families. Although the participants still need to speak and read in English, we expect that we will be able to recruit a number of caregivers who represent more vulnerable groups. To address and acknowledge this as a potential limitation, we have added this to our limitation section as follows: Lines 668-670 (tracked changes version): our study population will represent specific sub-populations within an English-speaking Canadian context. Therefore, our results will not necessarily be generalizable to other populations such as non-English speaking caregivers within Canada or elsewhere globally.*

- not asking parents to fill in a rating scale of their child's behavioral problems, as these parents are the ones who get the most out of the programs as they are more motivated.

*Although we agree with the reviewer, given we are not currently evaluating a program and that we are attempting to limit the length of the survey, we have not included a child behaviour measure. We have added this as a limitation to the study. Please see Lines 662-665:* *Although measures of child behaviour problems are typically collected in parenting program evaluations, to minimize participant burden, we did not include such a measure in the current study. Future research examining the effectiveness of the PLH materials should include a range of relevant outcomes, including child functioning.*

7. PLOS authors have the option to publish the peer review history of their article ([what does this mean?](https://journals.plos.org/plosone/s/editorial-and-peer-review-process#loc-peer-review-history)). If published, this will include your full peer review and any attached files.

If you choose “no”, your identity will remain anonymous but your review may still be made public.

**Do you want your identity to be public for this peer review?** For information about this choice, including consent withdrawal, please see our [Privacy Policy](https://www.plos.org/privacy-policy).

Reviewer #1: No

Reviewer #2: **Yes:** Maria Filomena Ribeiro da Fonseca Gaspar

[NOTE: If reviewer comments were submitted as an attachment file, they will be attached to this email and accessible via the submission site. Please log into your account, locate the manuscript record, and check for the action link "View Attachments". If this link does not appear, there are no attachment files.]

While revising your submission, please upload your figure files to the Preflight Analysis and Conversion Engine (PACE) digital diagnostic tool, <https://pacev2.apexcovantage.com/>. PACE helps ensure that figures meet PLOS requirements. To use PACE, you must first register as a user. Registration is free. Then, login and navigate to the UPLOAD tab, where you will find detailed instructions on how to use the tool. If you encounter any issues or have any questions when using PACE, please email PLOS at [figures@plos.org](mailto:figures@plos.org). Please note that Supporting Information files do not need this step.
