# Supplementary material for: CHAMPP4KIDS: Mixed methods study protocol to evaluate acceptability and feasibility of Parenting for Lifelong Health materials in a Canadian context
Source: PLoS One. 2024 Mar 7;19(3):e0298156. doi: 10.1371/journal.pone.0298156 (PMC10919627; doi:10.1371/journal.pone.0298156)
Supplement: Attachment — Submitted filename: PlosOne Response to Reviewers.docx [file pone.0298156.s002.docx]

Response to Reviewers

1. Does the manuscript provide a valid rationale for the proposed study, with clearly identified and justified research questions?

The research question outlined is expected to address a valid academic problem or topic and contribute to the base of knowledge in the field.

Reviewer #1: Yes

Reviewer #2: Yes

2. Is the protocol technically sound and planned in a manner that will lead to a meaningful outcome and allow testing the stated hypotheses?

The manuscript should describe the methods in sufficient detail to prevent undisclosed flexibility in the experimental procedure or analysis pipeline, including sufficient outcome-neutral conditions (e.g. necessary controls, absence of floor or ceiling effects) to test the proposed hypotheses and a statistical power analysis where applicable. As there may be aspects of the methodology and analysis which can only be refined once the work is undertaken, authors should outline potential assumptions and explicitly describe what aspects of the proposed analyses, if any, are exploratory.

Reviewer #1: Yes

Reviewer #2: Yes

3. Is the methodology feasible and described in sufficient detail to allow the work to be replicable?


Descriptions of methods and materials in the protocol should be reported in sufficient detail for another researcher to reproduce all experiments and analyses. The protocol should describe the appropriate controls, sample size calculations, and replication needed to ensure that the data are robust and reproducible.

Reviewer #1: Yes

Reviewer #2: Yes

4. Have the authors described where all data underlying the findings will be made available when the study is complete?

The [PLOS Data policy](https://journals.plos.org/plosone/s/materials-and-software-sharing) requires authors to make all data underlying the findings described in their manuscript fully available without restriction, with rare exception, at the time of publication. The data should be provided as part of the manuscript or its supporting information, or deposited to a public repository. For example, in addition to summary statistics, the data points behind means, medians and variance measures should be available. If there are restrictions on publicly sharing data—e.g. participant privacy or use of data from a third party—those must be specified.

Reviewer #1: Yes

Reviewer #2: No

Thank you for this comment. A data availability statement has now been added to the manuscript – please see lines 643-645.

5. Is the manuscript presented in an intelligible fashion and written in standard English?

PLOS ONE does not copyedit accepted manuscripts, so the language in submitted articles must be clear, correct, and unambiguous. Any typographical or grammatical errors should be corrected at revision, so please note any specific errors here.

Reviewer #1: Yes

Reviewer #2: Yes

6. Review Comments to the Author

Please use the space provided to explain your answers to the questions above and, if applicable, provide comments about issues authors must address before this protocol can be accepted for publication. You may also include additional comments for the author, including concerns about research or publication ethics.

You may also provide optional suggestions and comments to authors that they might find helpful in planning their study.


(Please upload your review as an attachment if it exceeds 20,000 characters)

Reviewer #1: The authors addressed all comments and I believe the protocol manuscript has been strengthened. This is minor, but the authors may consider how potential participants might interpret the question "are you a member of a racialized group?" - I work with a similar population and I believe parents may not fully understand the question. Therefore, it might be helpful to have some prompts for the RAs. Finally, although I understand the rationale for not including a child behavior measure due to length of survey, I wonder if the investigators considered the Strengths and Difficulties Questionnaire - it's only 25 items and takes less than 5 minutes to complete and may be a good addition.

We appreciate your suggestion to include prompts for the question "are you a member of a racialized group?" on our online screening page https://www.champp4kids.com/caregivers. While we recognize the potential for ambiguity, our decision not to modify the screener further stems from a balance between ensuring clarity and minimizing the risk of introducing leading cues. We believe that our current approach maintains a level of neutrality and allows participants to self-identify in a manner they find most fitting. However, your feedback will be considered for future refinements as we continually strive to enhance the quality of our research.

Also, thank you for your insightful comment regarding the omission of a child behavior measure due to survey length constraints. We appreciate your suggestion of the Strengths and Difficulties Questionnaire. While we did not include it in the current study, we acknowledge its potential value. Your input will be noted as a limitation in the protocol manuscript, and we will consider it for future directions in our research.

Reviewer #2: p1 Line 26: Frances Gardner should replace Frances Gardiner
p.3 line 88 The link for the reference nº14 isn’t working

Thank you for this edit. The correction to this professor’s name has been corrected.

Thank you for this comment. We have updated the link in the reference section, so it is searchable and functional.

7. PLOS authors have the option to publish the peer review history of their article ([what does this mean?](https://journals.plos.org/plosone/s/editorial-and-peer-review-process#loc-peer-review-history)). If published, this will include your full peer review and any attached files.


If you choose “no”, your identity will remain anonymous but your review may still be made public.


**Do you want your identity to be public for this peer review?** For information about this choice, including consent withdrawal, please see our [Privacy Policy](https://www.plos.org/privacy-policy).

Reviewer #1: No

Reviewer #2: **Yes:** Maria Filomena Ribeiro da Fonseca Gaspar

[NOTE: If reviewer comments were submitted as an attachment file, they will be attached to this email and accessible via the submission site. Please log into your account, locate the manuscript record, and check for the action link "View Attachments". If this link does not appear, there are no attachment files.]

While revising your submission, please upload your figure files to the Preflight Analysis and Conversion Engine (PACE) digital diagnostic tool, <https://pacev2.apexcovantage.com/>. PACE helps ensure that figures meet PLOS requirements. To use PACE, you must first register as a user. Registration is free. Then, login and navigate to the UPLOAD tab, where you will find detailed instructions on how to use the tool. If you encounter any issues or have any questions when using PACE, please email PLOS at [figures@plos.org](mailto:figures@plos.org). Please note that Supporting Information files do not need this step.

*In compliance with data protection regulations, you may request that we remove your personal registration details at any time.* [*(Remove my information/details)*](https://www.editorialmanager.com/pone/login.asp?a=r)*. Please contact the publication office if you have any questions.*
